# Endoscopic submucosal dissection for resection of giant adenoid cystic carcinoma of the esophagus

**DOI:** 10.1055/a-2346-4863

**Published:** 2024-07-15

**Authors:** Yu Xia, Jian Li, Guanzhang Wen, Qiqi Zhang, Jingjing Yao, Zhiqiang Zhang

**Affiliations:** 1 The Second Department of Gastroenterology, The First Affiliated Hospital of Xinjiang Medical University, Urumqi, China

A 53-year-old man presented with progressive dysphagia and retrosternal pain. A gastroscope revealed a giant pronounced protrusion lesion with a thick pedicle in the lower esophagus measuring approximately 60 × 25 mm. Further refinement via computed tomography (CT) indicated an occupying lesion in the distal esophagus with no evidence of metastatic disease present; this led to the suggestion of performing an endoscopic resection

The surgery was performed under general anesthesia with endotracheal intubation ( Video 1 ) involving submucosal injection of saline with diluted methylene blue, and the use of a DualKnife for the endoscopic mucosal dissection. Hemostatic forceps were employed intraoperatively to control bleeding. Postoperative treatment with sucralfate suspension was given for 4 weeks to promote mucosal recovery. Histology revealed a solid-type adenoid cystic carcinoma with ulceration affecting the submucosa with clean margins obtained from the resection.

Successful endoscopic resection of a giant adenoid cystic carcinoma of the esophagus.Video 1

At a 3-month follow-up, the patientʼs sensation of a foreign body during ingestion had resolved completely. Gastroscopy indicated white uniform scarring in the esophagus without luminal narrowing or residual tumor, and a repeat CT scan showed no signs of disease recurrence or metastasis.

Adenoid cystic carcinoma is common in salivary glands but rare in the esophagus, with the chief symptom being progressive dysphagia 1 . There is no standard treatment method established for esophageal adenoid cystic carcinoma, with radical resection being the preferred therapeutic option 2 . Postoperative radiotherapy is advised if the surgical margins are positive. Herein, we report a rare case of esophageal adenoid cystic carcinoma detected as a giant esophageal tumor through endoscopic examination and managed with endoscopic submucosal dissection leading to a radical resection without metastasis.

Endoscopy_UCTN_Code_CCL_1AB_2AC

## Notes

Endoscopy E-Videos https://eref.thieme.de/e-videos
*E-Videos* is an open access online section of the journal *Endoscopy* , reporting on interesting cases and new techniques in gastroenterological endoscopy. All papers include a high-quality video and are published with a Creative Commons CC-BY license. Endoscopy E-Videos qualify for HINARI discounts and waivers and eligibility is automatically checked during the submission process. We grant 100% waivers to articles whose corresponding authors are based in Group A countries and 50% waivers to those who are based in Group B countries as classified by Research4Life (see: https://www.research4life.org/access/eligibility/ ). This section has its own submission website at https://mc.manuscriptcentral.com/e-videos .
